# LPS Induces Opposing Memory-like Inflammatory Responses in Mouse Bone Marrow Neutrophils

**DOI:** 10.3390/ijms22189803

**Published:** 2021-09-10

**Authors:** Trim Lajqi, Maylis Braun, Simon Alexander Kranig, David Frommhold, Johannes Pöschl, Hannes Hudalla

**Affiliations:** 1 Department of Neonatology, Heidelberg University Children’s Hospital, D-69120 Heidelberg, Germany; trim.lajqi@med.uni-heidelberg.de (T.L.); maylis.braun@med.uni-heidelberg.de (M.B.); simon.kranig@med.uni-heidelberg.de (S.A.K.); David.Frommhold@klinikum-memmingen.de (D.F.); johannes.poeschl@med.uni-heidelberg.de (J.P.); 2 Klinik für Kinderheilkunde und Jugendmedizin, D-87700 Memmingen, Germany

**Keywords:** neutrophils, LPS, priming, trained sensitivity, tolerance, migration, phagocytosis

## Abstract

A growing body of evidence suggests that innate immune cells can respond in a memory-like (adaptive) fashion, which is referred to as trained immunity. Only few in vivo studies have shown training effects in neutrophils; however, no in vitro setup has been established to study the induction of trained immunity or tolerance in neutrophils by microbial agents. In light of their short lifespan (up to 48 h), we suggest to use the term trained sensitivity for neutrophils in an in vitro setting. Here, we firstly describe a feasible two-hit model, using different doses of lipopolysaccharide (LPS) in bone marrow neutrophils. We found that low doses (10 pg/mL) induce pro-inflammatory activation (trained sensitivity), whereas priming with high doses (100 ng/mL) leads to suppression of pro-inflammatory mediators such as TNF-α or IL-6 (tolerance) (*p* < 0.05). On a functional level, trained neutrophils displayed increased phagocytic activity and LFA-1 expression as well as migrational capacity and CD11a expression, whereas tolerant neutrophils show contrasting effects in vitro. Mechanistically, TLR4/MyD88/PI3Ks regulate the activation of p65, which controls memory-like responses in mouse bone marrow neutrophils (*p* < 0.05). Our results open a new window for further in vitro studies on memory-like inflammatory responses of short-lived innate immune cells such as neutrophils.

## 1. Introduction

The innate immune system is continuously challenged by several intracellular and extracellular stressors, resulting in a variety of pro- or anti-inflammatory responses. Innate immunity has long been considered as merely primitive due to a lack of immunological memory, which is classically only attributed to the acquired immune system. However, studies in different myeloid cells (i.e., monocytes, macrophages, natural killer (NK) cells), microglia, or non-immune cells challenge this notion and highlight the ability of innate immune cells to develop non-specific memory-like inflammatory responses to protect against secondary infections (trained immunity) [1,2,3,4,5,6].

Trained immunity is portrayed by an exacerbated immune response characterized with increased levels of pro-inflammatory mediators (such as TNF-α, IL-6, IL-1β, ROS, and IL-12), whereas the opposing reaction of tolerance is marked by suppressed levels of pro-inflammatory responses in addition to enhanced anti-inflammatory properties (such as IL-10, arginase 1 (Arg-1)) [4,5,7,8]. Immune priming represents a process where prior exposure to sublethal pathogen dose or other stressors triggers elevated immune reactions (resistance) to a subsequent potentially lethal infection [9,10,11]. Pioneering studies disclosed a pathogen-associated molecular pattern (PAMP)-specific induction of trained immunity, where priming by β-glucan or bacillus Calmette–Guerin (BCG) after subsequent challenge by gram (–) bacterial lipopolysaccharide (LPS) triggers an exacerbated inflammatory response [1,12,13,14]. Later on, several studies performed in macrophages and microglia revealed a pathogen dose-dependent development of trained immunity by priming with low doses of LPS or tolerance by high doses of LPS, after subsequent challenge with the same or a different stressor [5,15,16,17]. The maturation state is yet another factor that may influence the induction of memory-like responses, and may explained the magnitude of priming [7,18,19]. Various seminal studies have shown that both memory-like inflammatory responses are accompanied by epigenetic reprogramming with resulting distinct changes in metabolism [8,20,21,22].

Neutrophils are among the first responder cells of the innate immunity that enter the site of infection. They are the most abundant type of immune cells and the largest group of polymorphonuclear cells [23,24,25]. As terminally differentiated cells, neutrophils harbor an armory of antimicrobial agents, such as hydrolytic enzymes and pro-inflammatory mediators, and produce reactive oxygen species (ROS) in order to protect against a variety of different viral, bacterial, as well as fungal infections [26,27,28]. Due to their short live-span, in vitro studies are challenging and neutrophils are not the first to come in mind when considering memory-like responses. However, there is evidence that neutrophils may possess memory-like capacity. For example, priming by TNF-α, IL-8, as well as microbiome-derived metabolites improves the recruitment and killing capacities of neutrophils [29,30,31,32,33]. Interestingly, recent reports highlight the important role of primed neutrophils by β-glucan and BCG, driving anti-tumoral immune responses characterized by altered cytokine levels and ROS production, as well as enhanced antimicrobial activities upon a second challenge [34,35]. These long-term effects have been attributed to the process of trained immunity which may occur in the bone marrow during granulopoiesis (central trained immunity), as well as in the peripheral blood (peripheral trained immunity) [36]. However, the translational potential of mouse findings should be handled with caution, since murine neutrophils do not precisely mimic human neutrophils in function, morphology, or physiology [37,38].

Our present study outlines a new in vitro setup to study trained sensitivity, and establishes the role of LPS in the induction of memory-like inflammatory responses in neutrophils. Bone marrow neutrophils from adult mice were primed by increasing doses of LPS, and inflammatory responses upon a second challenge by LPS were evaluated. Low-dose LPS-primed mouse bone marrow neutrophils in vitro display increased pro-inflammatory responses (trained sensitivity), whereas priming by higher doses of LPS triggers immune tolerance characterized by decreased levels of pro-inflammatory mediators and increased IL-10. Trained neutrophils displayed increased phagocytic activity as well as migratory effects, whereas tolerant neutrophils exhibited reduced antimicrobial activity leading to reduced recruitment in vitro. The implications of the LPS priming for the inflammatory response, cellular functions, and related signaling patterns will be discussed.

## 2. Results

### 2.1. LPS-Priming Triggers Opposing Inflammatory Responses, Trained Sensitivity and Tolerance, in Mouse Bone Marrow Neutrophils

**Figure 1:**
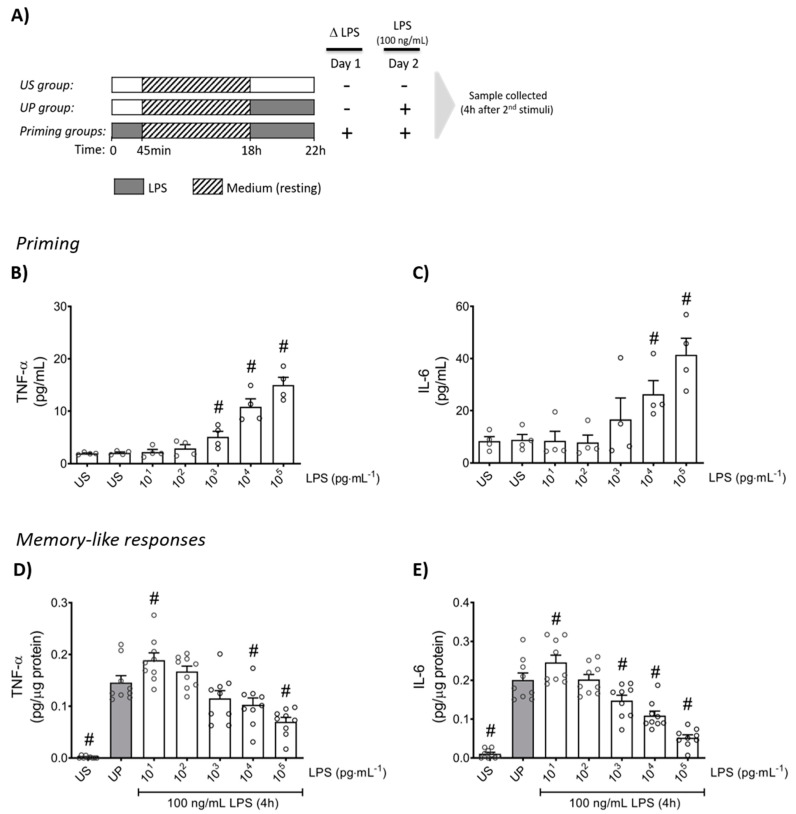
Cytokine responses of mouse bone marrow neutrophils primed by increasing doses of LPS. (**A**) Two-step stimulating schema used to induce memory-like inflammatory responses. Bone marrow neutrophils (1,000,000 cells/well) isolated from adult mice were stimulated with different doses of LPS (10 pg/mL to 100 ng/mL) on day 1 immediately after isolation for 45 min. Then, cells were washed and left in medium to rest for 18 h. On the next day (day 2), cells were re-stimulated with a fixed dose of LPS (100 ng/mL) for 4 h, followed by sample collection after the incubation time and processed for further analysis. Cytokine production of (**B**) TNF-α (n = 4) and (**C**) IL-6 (n = 4) by mouse bone marrow neutrophils stimulated (primed) with increasing doses of LPS analyzed by ELISA. (**D**,**E**) Opposing inflammatory responses of mouse bone marrow neutrophils primed by increasing doses of LPS, after a second challenge by a fixed dose LPS (100 ng/mL). Cytokine levels ((**D**): TNF-α, n = 9; **E**: IL-6, n = 9) assessed by ELISA (normalized to total protein concentration). Data are shown as scatter dot plots, mean + SEM, # *p* < 0.05, # significant differences vs. unstimulated condition (US) (**B**,**C**); # *p* < 0.05, # significant differences vs. unprimed condition (UP, gray column) (**D**,**E**).

Firstly, neutrophils were stimulated (primed) with increasing doses of LPS to verify the inflammatory response in a dose-dependent manner for the production of TNF-α and IL-6 (Figure 1B,C; *p* < 0.05). Upon a second challenge by a fixed dose of LPS (100 ng/mL), primed neutrophils expressed a distinct biphasic dose-response pattern for the production of pro-inflammatory cytokines, TNF-α and IL-6, exhibiting significantly increased levels by low-dose priming (*p* < 0.05), especially 10 pg/mL LPS, whereas high-dose (≥1 ng/mL) primed neutrophils showed markedly decreased cytokine amounts compared to unprimed state of neutrophils (Figure 1D,E; *p* < 0.05).

**Figure 2:**
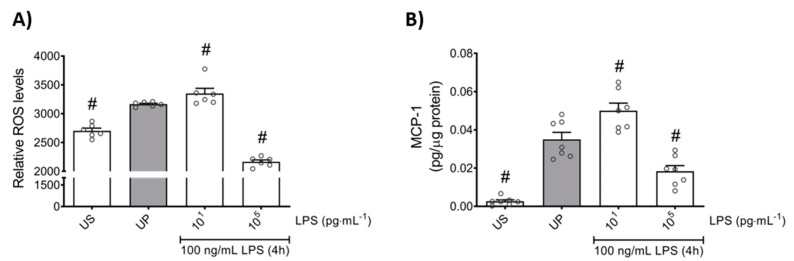
Effects of low- and high-dose LPS priming on ROS production and MCP-1 release in bone marrow neutrophils. Cells were primed with different doses of LPS (low-dose: 10 pg/mL; high-dose: 100 ng/mL) and re-challenged on day 2 with a fixed dose LPS (100 ng/mL) as described previously. (**A**) ROS levels (n = 6) were assessed by the 2′,7′–dichlorofluorescin diacetate (DCFDA) assay, whereas the production of (**B**) MCP-1 (n = 7) was measured using ELISA (normalized to total protein concentration). Data are shown as scatter dot plots, mean + SEM, # *p* < 0.05, # significant differences vs. unprimed condition (UP, gray column).

In order to further elaborate the effects of the LPS dose in the induction of memory-like responses in bone marrow neutrophils, we examined the production of ROS and monocyte chemoattractant protein-1 (MCP-1) as important mediators of inflammation and neutrophil recruitment. We found that both mediators are increased in neutrophils primed by 10 pg/mL (low-dose) LPS upon a second stimulation by LPS, revealing trained sensitivity responses compared to the unprimed state (Figure 2; *p* < 0.05). In contrast, priming by 100 ng/mL (high-dose) LPS triggered suppressed levels of ROS as well as MCP-1 upon a second challenge by LPS, displaying tolerance responses of mouse bone marrow neutrophils (Figure 2; *p* < 0.05).

**Figure 3:**
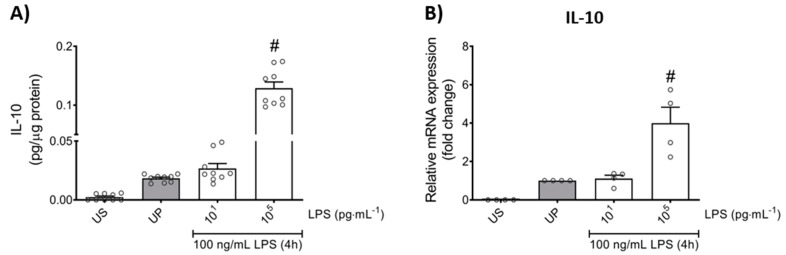
Effects of low- and high-dose LPS priming on the production of IL-10 as anti-inflammatory cytokine in mouse bone marrow neutrophils. Cells were primed with different doses of LPS (low-dose: 10 pg/mL; high-dose: 100 ng/mL) and re-challenged on day 2 with a fixed dose LPS (100 ng/mL) as described. Supernatants and RNA samples were collected 4 h after the second challenge. Production of (**A**) IL-10 (n = 9) was measured using ELISA (normalized to total protein concentration), whereas (**B**) gene expression of IL-10 (n = 4) by real-time PCR normalized to glyceraldehyde-3-phosphate dehydrogenase (GAPDH) representing relative values to unprimed state (assigned as 1.0). Data are shown as scatter dot plots, mean + SEM, # *p* < 0.05, # significant differences vs. unprimed condition (UP, gray column).

Further, we asked whether the anti-inflammatory response, assessed by IL-10 production, is equally affected by low- and high-dose LPS priming. Our results show that the anti-inflammatory cytokine IL-10 did not exhibit any changes by low-dose priming, but significantly displayed enhanced levels by high-dose priming (Figure 3; *p* < 0.05). These data about the anti-inflammatory pattern of neutrophils outline a contrary reaction to the pro-inflammatory status shown above in response to distinct priming doses of LPS.

Next, we asked whether the self-release of pro-inflammatory cytokines, such as TNF-α and IL-6, during the resting period of cells may account for the development of trained sensitivity and tolerance in neutrophils. We found that the release of TNF-α and IL-6 during the resting time remained unchanged (Supplementary Figure S1). Interestingly, the production of IL-6 was higher than the production of TNF-α. Moreover, we analyzed the viability of mouse bone marrow neutrophils during the experimental procedure and further measured cytotoxicity to elaborate any cytotoxic effects arising from LPS as a stressing agent. Our data show no significant changes for cell viability as well as cytotoxicity of our cells (Supplementary Figure S2).

### 2.2. LPS-Priming Is Mainly Promoted by TLR4/PI3K Activation of p65

**Figure 4:**
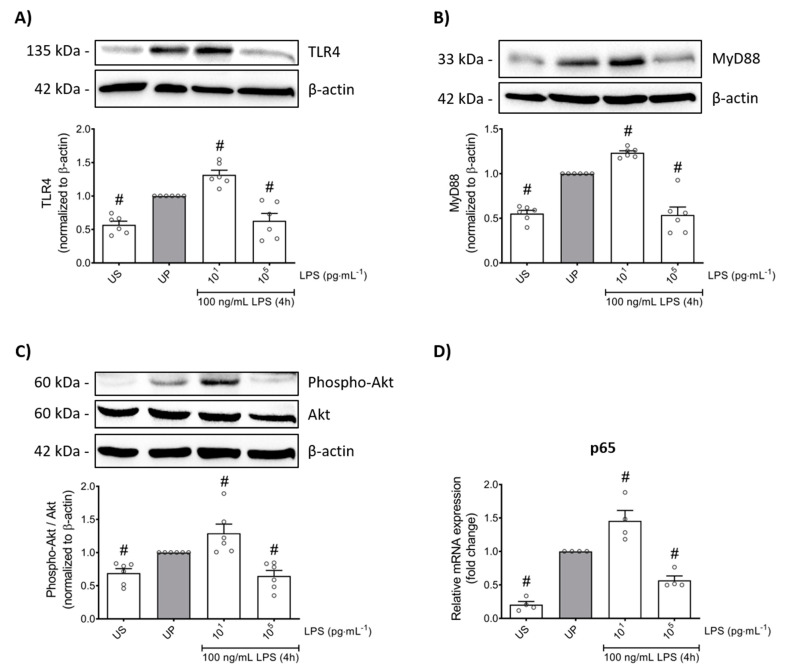
LPS-induced alterations of the TLR4/MyD88/PI3Ks triangle triggers opposing reactions in p65 transcription, promoting either trained sensitivity or tolerance responses in mouse bone marrow neutrophils. Cells were primed with different doses of LPS (low-dose: 10 pg/mL; high-dose: 100 ng/mL) and re-challenged on day 2 with a fixed dose LPS (100 ng/mL) as described. Lysates were collected 4 h after the second stimulation by LPS and the protein expression of (**A**) TLR4 (n = 6), (**B**) MyD88 (n = 6) and (**C**) phospho-Akt were assayed by Western blotting and quantified (unprimed cells assigned as 1.0). RNA samples were collected also 4 h after the second stimulation and analyzed for the gene expression of (**D**) p65 by real-time PCR normalized to GAPDH representing relative values to unprimed state (assigned as 1.0). Data are shown as scatter dot plots, mean + SEM, # *p* < 0.05, # significant differences vs. unprimed condition (UP, gray column).

We next asked for signaling mediators potentially accounting for the change in pro-inflammatory mediators such as TNF-α, IL-6, MCP-1, and ROS, as well as the anti-inflammatory cytokine IL-10, in response to low- and high-dose LPS priming in mouse bone marrow neutrophils. Protein expression analysis of toll-like receptor 4 (TLR4) as recognition receptor for LPS, and its adaptor protein myeloid differentiation primary response protein 88 (MyD88) revealed similarly dose-response arrangements, where low-dose priming triggered trained sensitivity characterized by an upregulation of TLR4/MyD88, whereas tolerance development induced by high-dose priming was characterized with downregulated protein levels of related proteins (Figure 4A,B; *p* < 0.05).

Based on previous reports on role of phosphoinositide 3-kinases (PI3Ks) as crucial proteins for the induction of memory-like responses, we then assessed the protein expression of its downstream mediator protein kinase B (Akt). Comparable dose-dependent findings were found, whereas trained neutrophils expressed increased Akt phosphorylation, different from tolerant neutrophils that displayed decreased phosphorylation levels of Akt (Figure 4C; *p* < 0.05). Concordant responses were observed for the nuclear factor kappa B (NFκB) subunit p65 (Figure 4D; *p* < 0.05).

Altogether, our data show that low-dose LPS-induced trained sensitivity is mainly promoted by the signaling triangle of TLR4/MyD88/PI3Ks triggering the activation of p65, resulting in increased production of the pro-inflammatory mediators such as cytokines (TNF-α, IL-6), chemokine (MCP-1), and ROS. In contrast, high-dose LPS-induced tolerance in mouse bone marrow neutrophils displayed diminished levels of p65 mainly due to the suppressed signaling mechanisms of TLR4/MyD88/PI3Ks, resulting in diminished levels of the related pro-inflammatory mediators and an increased anti-inflammatory response (IL-10).

### 2.3. Trained Sensitivity and Tolerance Alter the Migratory and Phagocytic Activity of Mouse Bone Marrow Neutrophils

**Figure 5:**
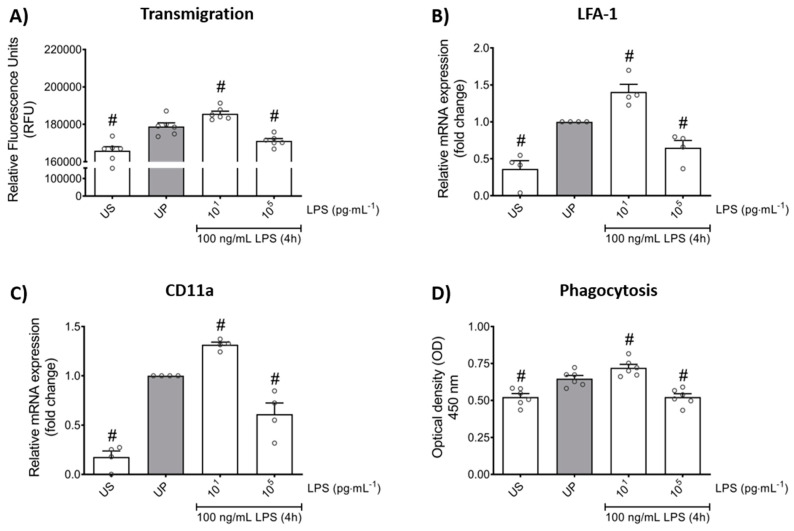
Effects of low- and high-dose LPS priming on the migratory effects and phagocytic activity of mouse bone marrow neutrophils in vitro. Cells were primed with different doses of LPS (low-dose: 10 pg/mL; high-dose: 100 ng/mL) and re-challenged on day 2 with a fixed dose LPS (100 ng/mL), as described. (**A**) Transmigration of neutrophils (n = 6) was analyzed by the CytoSelect 96-well Cell Migration Assay, as described (check Materials and methods, Section 4.8) and data were expressed as relative fluorescence units. RNA samples were collected 4 h after the second stimulation by LPS (100 ng/mL) and analyzed for (**B**) LFA-1 (n = 4) and (**C**) CD11a (n = 4) by real-time PCR normalized to GAPDH representing relative values to unprimed state (assigned as 1.0). (**D**) Phagocytic activity of mouse bone marrow neutrophils (n = 6) was assessed using a CytoSelect 96-Well Phagocytosis Assay kit (E. coli Substrate) and data were expressed as optical density (further check Material and methods, Section 4.9). Data are shown as scatter dot plots, mean + SEM, # *p* < 0.05, # significant differences vs. unprimed condition (UP, gray column).

In order to verify whether LPS-induced memory-like responses also translate to altered cellular functions in mouse bone marrow neutrophils, we assessed the transmigratory effects as well as the neutrophil phagocytic activity in vitro. Our cell culture studies showed that LPS-induced trained sensitivity by low-dose priming resulted in a significant increase in the migratory effects followed by increased expression of its related β_2_ integrin, important for neutrophil recruitment, and lymphocyte function-associated antigen 1 (LFA-1) (Figure 5A,B; *p* < 0.05). In contrast, high-dose priming by LPS led to decreased migratory properties and LFA-1 expression (*p* < 0.05). We then verified a similar pattern for the expression of the α chain of LFA-1, the CD11a which is highly expressed in leukocytes and represents an important factor of cell recruitment (Figure 5C; *p* < 0.05).

Lastly, we asked about the role of LPS priming on the phagocytic activity of neutrophils. Our in vitro studies revealed that LPS treatment alone triggers increased phagocytic activity in neutrophils (Figure 5D; *p* < 0.05). Comparable to migratory effects, trained neutrophils exhibited increased phagocytic activity whereas tolerant neutrophils were characterized by suppressed phagocytosis.

Taken together, our data demonstrate the importance of LPS priming dose in the development of memory-like inflammatory responses that alter cellular functions, such as migration and phagocytosis, in mouse bone marrow neutrophils in vitro.

## 3. Discussion

Neutrophils as a first line of innate immune cells are an essential component of the host defense against invading organisms. They have long been considered as mere effector cells that express their repertoire of antimicrobial agents, such as hydrolytic enzymes, ROS, cytokines, and chemokines, in a simple reactive fashion in response to conserved molecules known as PAMPs present by infectious microorganisms [24,27,39]. These microbial components, PAMPs, are detected by several germline-encoded host sensors labeled as pattern recognition receptors (PRRs) that trigger the activation of a cascade of signaling proteins resulting in the release of a wide range of inflammatory components as a resistance mechanism of neutrophils [40,41,42,43]. Neutrophils are key phagocytic players primarily characterized by their antimicrobial potential, but more recent studies have shown that neutrophils may also promote repairing mechanisms by the release of the anti-inflammatory mediator IL-10 [39,44,45,46]. It is, however, important to realize the limitations of translating mouse neutrophil findings to human neutrophil populations. Gene expression patterns in neutrophils which govern major immune functions such as activation, development, and pathogen responses greatly diverge between species [37,47,48]. In particular, the cytokine and chemokine production, which serves as important determinants of memory-like responses, differ drastically between species and cell populations [37,47].

The notion of memory-like responses in innate immune cells has generally challenged the well-known concept of dichotomy between innate and acquired immunity. Initial studies disclosed the stressor-dependent induction of both trained immunity and tolerance in monocytes and macrophages, where priming by β-glucan or BCG triggers training, distinct from LPS-priming that results in tolerance upon a second infection insult [3,4,13,14]. Subsequently, dose-dependent priming effects mediating both memory-like responses in innate immune cells were established [5,15,49,50,51]. Moreover, studies from our group also revealed the magnitude of priming and the cellular maturation state as important factors in the development of memory-like patterns [7]. Both of memory-like responses are accompanied by epigenetic reprogramming regulated by the methylation or acetylation of H3 and H4 histones (i.e., H3K4me3, H3K27ac, H3K9me3, H4K20me3), with resulting distinct changes in metabolism such as increased glycolysis, glutaminolysis, or accumulation of fumarate during trained immunity or increased itaconate production and fatty acid oxidation during tolerance [8,14,20,21,22,52,53,54].

The current study provides supporting evidence for a dose-dependent induction of training and tolerance in neutrophils in vitro. We show, for the first time, that the patterns of memory-like responses in mouse bone marrow neutrophils can be differentially regulated by the pathogen dose, specifically LPS. Training can occur both in the bone marrow or in the peripheral blood [36]. We chose mouse bone marrow neutrophils for our study in order to reduce the confounder of circulating endotoxins. As expected, a single stimulation by increasing doses of LPS triggered dose-dependent production of pro-inflammatory cytokines. Challenging the cells with a second stimulation after 18 h of resting showed a biphasic dose response, similar to hormetic reactions where low-dose LPS (10 pg/mL) displayed enhanced levels of pro-inflammatory cytokines (trained sensitivity), distinct from high-dose LPS (≥1 ng/mL) priming that resulted in suppressed production of cytokines (tolerance). A previous study demonstrated similar findings in myeloid cells (monocytes), primed continuously by LPS [15]. Similarly, microglia expressed comparable LPS-dose patterns where continuously priming with low or ultra-low doses in vitro upon a second challenge provokes exacerbated pro-inflammatory reactions [5,7,16,55]. In line with our findings, tolerance development was clearly described by high-dose priming with LPS.

ROS is known to be a critical antimicrobial component of neutrophils promoting resistance mechanisms [39]. Furthermore, MCP-1 is well known to orchestrate the migratory actions during physiological and pathological circumstances in leukocytes as well as promoting the killing abilities of myeloid cells [56,57]. Trained sensitivity-induced reactions in mouse bone marrow neutrophils are supported largely by ROS and MCP-1 production leading to increased phagocytic activity and recruitment. Interestingly, tolerant neutrophils displayed suppressed levels of ROS and MCP-1 and increased production of IL-10. Our data are in line with the recent work published by Kalafati et al., showing that trained neutrophils in vivo promote anti-tumoral effects in a ROS-dependent manner [34]. Moreover, suppressed levels of pro-inflammatory mediators escorted by increased IL-10 levels support the resolution of inflammation and drive repairing mechanisms of neutrophils, as reported previously [39,44,45,46]. IL-10 is an important anti-inflammatory mediator not only pivotal for the establishment of tolerance responses by inhibiting the production of pro-inflammatory cytokines and chemokines, but also playing a regulatory role in neutrophils and protecting the host from exaggerated inflammatory responses to pathogens [58,59]. The non-significant IL-10 production by low-dose LPS priming could be explained by the findings of Yuan et al., which show that low-dose LPS-induced training may be responsible for the removal of B lymphocyte-induced maturation protein-1 (Blimp-1), thus dismissing the development of adequate anti-inflammatory response and favoring the inflammatory adaptation [15]. During the resting period, mouse bone marrow neutrophils did not display any dose-dependent significant alterations, but the levels of IL-6 tended to be higher than TNF-α. This might be explained by chromatin modulation occurring in the IL-6 regulatory genes mainly promoted by endogenous TNF-α [60,61,62].

LPS as a prominent PAMP is a potent endotoxin that is sensed by the PRR receptor toll-like receptor (TLR) 4 [63,64]. Earlier studies reported that extremely low LPS doses (femtomolar) may trigger the activation of myeloid differentiation factor-2 (MD-2)/TLR4 complex, most probably due to the increased sensitivity driven by CD14 [65,66,67,68]. Activation of TLR4 results in a cascade of signaling events promoting the release of inflammatory mediators mainly by a concordant activation of NFκB, especially p65 [69,70]. Our data show similar activation patterns, with trained neutrophils displaying activation of transcription factor p65 triggered by TLR4/MyD88, as opposed to tolerant neutrophils displaying downregulated levels of TLR4/MyD88 followed by suppressed transcription of p65. Previous reports also revealed that the family of PI3K signaling proteins are prone to mediate inflammatory responses of low-dose LPS in myeloid cells [71,72,73,74]. Similar effects of Akt-PI3K as intermediate mediators promoting the activation of p65 were observed in our study for mouse bone marrow neutrophils.

The pro-inflammatory activity of trained mouse bone marrow neutrophils exhibiting increased migratory activities and phagocytosis in vitro is particularly regulated by the TLR4/PI3K pathway. Complementary to our findings, Zhang et al. showed that neutrophil recruitment and phagocytic activity are guarded mainly by microbiota-induced signaling events driven by the TLR/MyD88 pathway [74,75,76]. Induction of tolerance by suppression of TLR4/PI3K/p65 reduces production of pro-inflammatory cytokines, migration, and phagocytosis, but on the other hand supports increased IL-10 production. These findings are in line with a recent study on LPS-induced tolerance in neutrophils by *Porphyromonas gingivalis*, demonstrating that altered cytokine responses due to endotoxin favor limited excessive inflammatory responses in order to facilitate survival [77].

We show that increased migratory effects in trained mouse bone marrow neutrophils are accompanied by enhanced expression of the leukocyte β_2_ integrin LFA-1 as well as its α chain CD11a, which are key molecules of the neutrophil recruitment cascade during inflammation as formerly outlined by numerous reports [78,79,80,81,82,83].

Taken together, the induction of opposing immune reactions shaped by LPS may serve as a binary switch to either efficiently eradicate pathogens (training as a resistance mechanism), or to support the resolution of inflammation (tolerance as persistence response) [25,84]. Recent studies on trained neutrophils, which may alter the diseases state or promote anti-tumoral activities, highlight the importance of memory-like responses during pathologies [34,35]. Our data transfer the concept of pathogen-induced, dose-dependent memory-like responses from macrophages to neutrophils. We further provide an in vitro assay to expand studies of adaptive elements in neutrophils. Whether these in vitro observations are of biological relevance in vivo remains unknown and warrants further investigations. We hope that our work on neutrophils will provoke further discussions and extended investigations about the physiological relevance considering the pivotal role of neutrophils in health and disease.

## 4. Materials and Methods

### 4.1. Animals and the Isolation of Mouse Bone Marrow Neutrophils

Adult (3–6 months old) locally bred mice, from a C57Bl/6J [Charles River (Sulzfeld, Germany)] breeder, were used for the isolation of bone marrow neutrophils. All mice were maintained at a 12-h light/dark cycle with ad libitum access to food and water at the Central Animal Facility of the University of Heidelberg, Germany. Experiments were carried out according to the guidelines from Directive 2010/63/EU of the European Parliament on the protection of animals used for scientific purposes and approved by the local authorities for animal welfare—Regierungspraesidium Karlsruhe, Germany (permission number for tissue and organ harvesting: Az T-02/20).

Murine bone marrow neutrophils were isolated from femurs and tibias, followed by the discontinuous Percoll (#17-0891-02, GE Healthcare) gradient, as described [85,86,87]. Neutrophils were harvested from the 64%/81% interface, washed in phosphate buffered saline (PBS), and cultivated in RPMI-1640 medium (#R8758, Sigma-Aldrich, St. Louis, MI, USA) supplemented with 10% heat-inactivated fetal bovine serum (FBS, #PB-FCS-EU-0500, PeloBiotech; endo-toxin-free and sterile-filtered), 1% penicillin/streptomycin, and 1% amphotericin B. The cell viability after isolation was greater than 95%, as assessed by the trypan blue (AppliChem, Darmstadt, Germany) exclusion test, and the purity of neutrophils was greater than 98%, as analyzed by microscopy using Hemacolor staining (Merck, Darmstadt, Germany) as previously shown [85,88].

### 4.2. Neutrophil Cell Stimulation Procedure

Immediately after isolation, neutrophils (1,000,000 cells/well) were primed according to the stimulation scheme depicted in Figure 1A. Cells were stimulated twice following a two-step protocol with an initial stimulation step (“priming”) with increasing doses of LPS (“first challenge”; 10 pg/mL—100 ng/mL for 45 min, respectively; E. coli serotype 055:B5 with obtained from InvivoGen (#tlrl-pb5lps), Toulouse, France) on day 1 immediately after isolation and incubated (37 °C; 5% CO_2_). Thereafter, cells were centrifuged and the medium was changed twice (“washing up”), and the cells were incubated overnight to rest at 37 °C and 5% CO_2_. On the next day (day 2), cells were re-stimulated (“second challenge”) with a fixed dose of LPS (100 ng/mL) for 4 h incubated at 37 °C and 5% CO_2_. RNAs and protein samples were collected 4 h after the second challenge with LPS and processed for further analysis. In parallel, cells cultivated only in the medium, i.e., the unstimulated (US) group, and cells stimulated once on day 2 for 4 h with a fixed dose of LPS (100 ng/mL), i.e., the unprimed (UP) group, were included as controls for measurements.

### 4.3. Antibodies

Primary antibodies, such as phospho-Akt (Ser473) (#9271), Akt (#9272), MyD88 (#4283), and TLR4 (#14358), were purchased from Cell Signaling (USA). The antibody against β-actin (#A5441) was obtained from Sigma Aldrich (St. Louis, MO, USA). Secondary HRP-coupled anti-rabbit and anti-mouse antibodies were purchased from Dianova (Hamburg, Germany).

### 4.4. SDS-PAGE Western Blotting

Cells were lysed using RIPA buffer containing 50 mM Tris/HCl pH 8, 150 mM NaCl, 1% (*v*/*v*) NP-40, 0.5% (*v*/*v*) Na-deoxycholate, 0.1% (*w*/*v*) SDS, 100 mg/mL Pefa-Block, 1 mg/mL Pepstatin A, 10 mM sodium vanadate, and 1 mg/mL Leupeptin. Samples were centrifuged (13.500× *g* for 30 min at 4 °C), and supernatants were mixed with 5× protein sample buffer (5% SDS, 33% glycerol, 25% β-mercaptoethanol, 83 mM Tris-HCl with pH to 6.8 and 0.1 mg/mL bromophenol blue) and heated for 5 min at 95 °C. Protein samples were separated on 10% polyacrylamide gel, transferred to a 0.45 μm polyvinylidenfluoride (PVDF) membrane, and then immunoblotted with above mentioned primary antibodies. Protein bands were detected by enhanced chemiluminescence reaction using ChemiDoc XRS+ camera (Bio-Rad Laboratories, Hercules, CA, USA). Quantification of the protein bands on the membrane was performed using the Image Lab Ver. 6.0.1 software (Bio-Rad Laboratories, Hercules, CA, USA).

### 4.5. Measurement of the Protein Concentration

Total protein concentration was determined using the Pierce™ 660 nm Protein Assay kit (#22662) from Thermo Fisher Scientific (Waltham, MA, USA). Ionic detergent compatibility reagent (IDCR) (#22663, Thermo Fischer Scientific, Waltham, MA, USA) was used in order to increase the detergent compatibility and reduce interference. Briefly, 10 μL of standard, sample, and blank in duplicates were plated in a 96-well plate, followed by the im-mediate addition of a 150-μL assay reagent supplemented with IDCR. Then, the plate was covered and left shaking for 1 min in a plate shaker. Afterwards, the plate was incubated for an additional 5 min at room temperature without shaking. Absorbance was measured at 660 nm using an iMark Microplate Reader (Bio-Rad Laboratories, Hercules, CA, USA). Protein concentration was then calculated based on the values of the standard curve.

### 4.6. Measurement of Cytokine and Chemokine Production

Cytokine and chemokine concentrations in supernatants were measured using commercial enzyme-linked immunosorbent assay (ELISA) kits for TNF-α (#430901), IL-6 (#431301), MCP-1 (#432701), and IL-10 (#431411) obtained from BioLegend (San Diego, CA, USA), as described [7]. The absorbance was read at 450 nm with a second reference wavelength at 570 nm in an iMark Microplate Reader (Bio-Rad Laboratories, Hercules, CA, USA). Cytokine levels of TNF-α, IL-6, MCP-1, and IL-10 after the second stimulation were normalized against the protein concentrations of each sample and depicted as pg/μg of total protein.

### 4.7. Measurement of ROS

ROS were measured using the DCFDA/H2DCFDA-Cellular ROS Assay Kit (#ab113851, Abcam, Cambridge, UK). The assay is based on the use of 2′,7′ –dichlorofluorescin diacetate (DCFDA, also known as H2DCFDA), a membrane-permeable fluorogenic dye that quantitatively measures hydroxyl, peroxyl, and other reactive oxygen species (ROS) in living cells. After diffusion into the cell, the DCFDA is deacetylated by cellular esterases to a non-fluorescent compound, which is later oxidized by ROS into 2′,7′ –dichlorofluorescein (DCF). DCF is highly fluorescent and is detected by fluorescence spectroscopy.

Briefly, mouse bone marrow neutrophils were seeded into black clear bottom 96-well plates (100,000 cells/well) and stimulated as described above. For measurement, the medium was aspirated and cells were resuspended in 100-μL DCFDA solution (20 μM) and incubated for 30 min at 37 °C protected from the light. Thereafter, cells were carefully washed twice with the 1× Buffer solution provided in the kit and resuspended in 1 ×Supplementary Buffer (supplemented with 10% FCS). The levels of intracellular ROS were detected by the fluorescence spectroscopy with excitation (Ex)/emission (Em) at 485 nm/535 nm using a PerkinElmer Wallac Victor3 1420 Multilabel Plate Reader (PerkinElmer Life and Analytical Sciences, Turku, Finland).

### 4.8. Transmigration/Chemotaxis Assay (In Vitro)

The transmigration/chemotaxis assay was performed using the CytoSelect 96-well Cell Migration Assay kit (#CBA-104, Cell Biolabs, San Diego, CA, USA; 3 µm, Fluorometric Format), according to the manufacturer’s instructions. Briefly, mouse bone marrow neutrophils were primed and then, followed by a second stimulation with LPS (100 ng/mL) the next day, as shown above in FCS-free medium. Next, 100 μL of the resuspended cells per well (10^6^ cells/mL) were placed into the 96-well plate upper chamber (membrane chamber; 3 μm polycarbonate membrane) and the membrane then was placed into the lower compartment (feeder tray) containing 150-μL medium supplemented with 10% FCS (as chemoattractant). After a 5-h incubation at 37 °C and 5% CO_2_, the remaining cell suspension from the inside the membrane chamber was carefully discarded and transferred immediately to a clean 96-well plate containing 150 µL of pre-warmed cell detachment solution. This plate was incubated for 30 min at 37 °C to completely dislodge the cells from the underside of the membrane chamber.

Thereafter, 75 µL of the detachment solution were combined with 75 µL of the media from the feeder tray and mixed well in a clear 96-well plate, followed by the addition of 50 µL 4× Lysis buffer/CyQuant GR dye solution to each well. The plate was then incubated for 20 min at room temperature. For the measurement, 150 µL of the mixture (containing 150 µL of migrated cells and 50 µL dye) was transferred to a black clear bottom 96-well plate for fluorescence measurement. Fluorescence was detected using a PerkinElmer Wallac Victor3 1420 Multilabel Plate Reader (PerkinElmer Life and Analytical Sciences, Turku, Finland) with Ex/Em at 485 nm/535 nm and expressed as relative fluorescence units (RFU).

### 4.9. Phagocytosis Assay (In Vitro)

The phagocytic activity of neutrophils was determined using CytoSelect 96-well Phagocytosis Assay kit (E. coli Substrate) (#CBA-222, Cell Biolabs, San Diego, CA, USA), according to the manufacturer’s instructions. Briefly, isolated mouse bone marrow neutrophils were stimulated, according to the depicted scheme shown above (Figure 1A) in a 96-well plate (100,000 cells/well). Then, 4 h after the second challenge by LPS (100 ng/mL), 10 µL of E. coli suspension was added to each well, mixed well, and the plate was incubated at 37 °C and 5% CO_2_ for 4 h. Afterwards, the plate was centrifuged (300 G for 5 min) and washed using ice cold 1× phosphate buffered saline (PBS) and cells were fixed using 100 µL/well fixation solution for 5 min at room temperature. Next, the plate was centrifuged (300 G for 5 min) and washed twice using 1× PBS. Then, 100 µL/well of 1× blocking solution was added and the plate was incubated at room temperature for 30 min in an orbital shaker. Thereafter, the plate was washed thrice using 1× PBS, and 100 µL/well of 1× permeabilization solution was added. The plate was incubated for 5 min at room temperature and, afterwards, it was washed twice using 1x PBS, followed by the process of initiating the reaction by adding a 100 µL/well substrate solution and incubated for 30 min at room temperature. Next, the reaction was stopped using stop solution (100 µL/well) and by placing the plate on an orbital plate shaker for 30 s. Absorbance was measured at 450 nm in the iMark Microplate Reader (Bio-Rad Laboratories, Hercules, CA, USA) and the data were given as optical density (OD) values.

### 4.10. Analysis of Cell Viability and Cytotoxicity

Cell viability was determined using the MTT assay. Murine bone marrow neutrophils were seeded into a 96-well plate (200,000 cell/well) and stimulated according to the stimulation scheme described above. Four hours after the second LPS stimulation, 10 µL/well of 0.5 mg/mL MTT (3-(4,5-dimethylthiazol-2-yl)-2,5-diphenyltetrazolium bromide) solution was added to the plate and incubated for 4 h at 37 °C and 5% CO_2_. Next, 100 µL of solubilization solution was added to each well and the plate was incubated overnight at 37 °C (5% CO_2_). The absorbance was measured at 570 nm using an iMark Microplate Reader (Bio-Rad Laboratories, Hercules, CA, USA) and data were shown as relative viability (UP assigned as 100%) (Supplementary Figure S2A).

In parallel, a second readout was performed to investigate the cytotoxicity of our stressor, LPS, in schema endured in our study using the Cell Cytotoxicity Assay kit (colorimetric; #ab112118, Abcam, Cambridge, UK). Isolated cells (10,000 cell/well) were seeded into a 96-well plate and stimulated as described above. Four hours after the second LPS stimulation, 20 µL of assay solution was added to each well and the plate was incubated initially for 30 s at room temperature in an orbital plate shaker. Thereafter, the plate was incubated at 37 °C and 5% CO_2_ for 4 h. The absorbance was measured at 570 nm using an iMark Microplate Reader (Bio-Rad Laboratories, Hercules, CA, USA) and data were shown as relative cytotoxicity (UP assigned as 100%) (Supplementary Figure S2B).

### 4.11. RNA Isolation and Real-Time qPCR

To determine gene expression levels, the total RNA was extracted from mouse bone marrow neutrophils 4 h after the 2nd stimulation with LPS (100 ng/mL) using QIAzol Lysis Reagent (#79306) purchased from Qiagen (Hilden, Germany) following the manufacturer instructions. RNA concentration and quality were checked using the Nanodrop DS-11 FX+ machine (DeNovix, Wilmington, DE, USA). During the whole procedure, RNase Away (#7003, Molecular BioProducts) solution was used to flush pipettes and other equipment in order to prevent any contamination with other RNases or DNAs. Complementary DNA (cDNA) was synthesized using RevertAid First Strand cDNA Synthesis kit (#K1612) from Thermo Fisher Scientific (Waltham, MA, USA). Real-time qPCR reaction was performed by using StepOnePlusTM real-time PCR System (Applied Biosystems, Waltham, MA, USA). The following primer pairs were used for this study: p65 forward: CTTCCTCAGCCATGGTACCTCT and p65 reverse: CAAGTCTTCATCAGCATCAAACTG, IL-10 forward: ACCAGCTGGACAACATACTGC and IL-10 reverse: TCACTCTTCACCTGCTCCACT, CD11a forward: AGATCGAGTCCGGACCCACAG and CD11a reverse: GGCAGTGATAGAGGCCTCCCG, LFA-1 forward: CTTGGACTTCCACTTCCACTTC and LFA-1 reverse: ACCTGGTAGACATGCTGGACTT, GAPDH forward: CATGGCCTTCCGTGTTTCCTA and GAPDH reverse: CCTGCTTCACCACCTTCTTGAT. GAPDH was used as housekeeping gene. Relative gene expression was calculated using the comparative C_T_ (2^−∆∆C^_T_) method [89].

### 4.12. Statistical Analysis

Graphs were prepared using GraphPad Prism 8.0.2 (GraphPad Software, San Diego, CA, USA), whereas statistical analysis was carried out using SigmaPlot Software Version 12.0 Build 12.0.0.182 (Systat Software GmbH, Erkrath, Germany). Data are presented as scatter dot plots, mean ± SEM. Comparison between experimental groups was performed using one-way analysis of variance (ANOVA). Post-hoc comparisons were performed by the Holm–Sidak test. *p* < 0.05 was considered significantly different.

## 5. Conclusions

Our study shows that the pathogenic stressor, LPS, may trigger the induction of memory-like responses in mouse bone marrow neutrophils in a dose-dependent manner. Low-dose priming triggers trained sensitivity characterized by a pro-inflammatory state, whereas high-dose priming drives toward a tolerant phenotype with an increased anti-inflammatory response. These distinct priming mechanisms translate into opposing cellular functions such as phagocytosis in mouse bone marrow neutrophils. Further investigations are necessary to explore the biological relevance of our in vitro findings.

## Data Availability

The data presented in this study are available on request from the corresponding author upon reasonable request.

## Supplementary Materials

The following are available online at https://www.mdpi.com/article/10.3390/ijms22189803/s1, Supplementary Figure S1: Cytokine production of TNF-α and IL-6 before second challenge by LPS. Supplementary Figure S2: Analysis of cell viability and cytotoxicity in primed bone marrow neutrophils.
