# Supplementary material for: LPS Induces Opposing Memory-like Inflammatory Responses in Mouse Bone Marrow Neutrophils
Source: Int J Mol Sci. 2021 Sep 10;22(18):9803. doi: 10.3390/ijms22189803 (PMC8469609; doi:10.3390/ijms22189803)
Supplement: Supplementary file 1 [file ijms-22-09803-s001.zip › ijms-1295274-supplementary.pdf]

Supplementary Figures

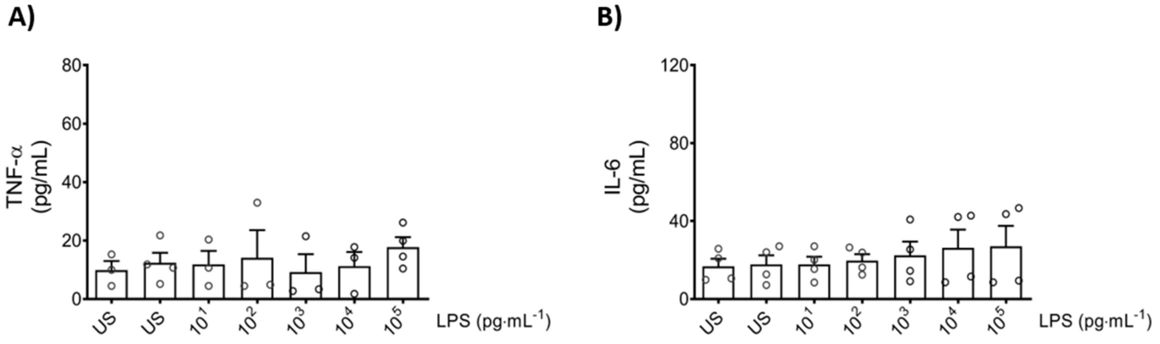

Supplementary Figure S1. Cytokine production of TNF-α and IL-6 before second challenge by LPS. Bone-marrow neutrophils (1.000.000 cells/well) isolated from adult mice were stimulated with different doses of LPS (10 pg/mL to 100 ng/mL) on day 1 immediately after isolation for 45 minutes. Thereafter, cells were washed up and left in medium to rest for 18 hours. Next day (day 2), before the second challenge with LPS (100 ng/mL), supernatants have been collected and the cytokine production for (**A**) TNF-α (n=3-4) and (**B**) IL-6 (n=4) were measured by ELISA. Data are shown as scatter dot plots, mean + SEM.

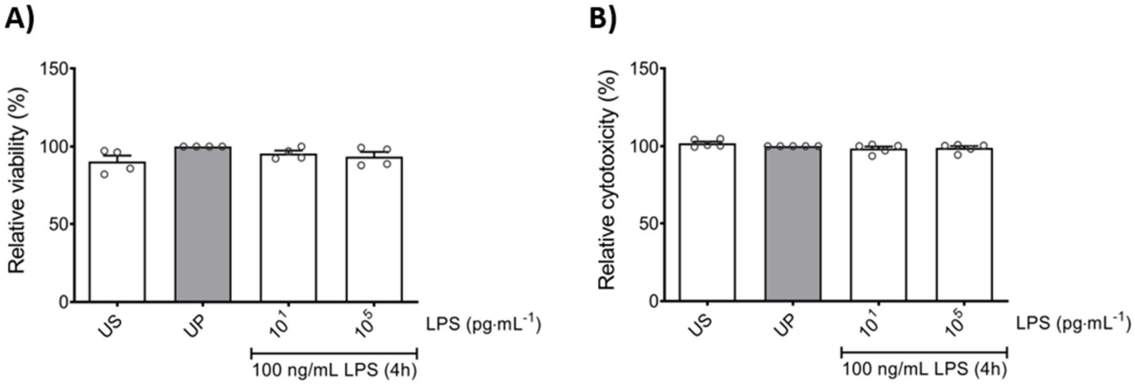

Supplementary Figure S2. Analysis of cell viability and cytotoxicity in primed bone-marrow neutrophils. Cells were primed with different doses of LPS (low-dose: 10 pg/mL; high-dose: 100 ng/mL) and re-challenged on day 2 with a fixed dose LPS (100 ng/mL) as described. The analysis of (**A**) cell viability (n=4) was performed by MTT assay, whereas the (**B**) cytotoxicity (n=5) of the stressor, LPS, was assessed using the commercial kit as described in the method section. Data are shown as scatter dot plots (UP group assigned as 100%), mean + SEM.
